# Optimizing the timing of painless gastroscope insertion: integrating advanced monitoring tools to enhance safety and efficacy: a narrative review

**DOI:** 10.3389/fmed.2026.1809637

**Published:** 2026-04-10

**Authors:** Chunyan Li, Hongmei Zhou, Shuai Kang, Yipan Wu, Li Hu, Yun Xiong

**Affiliations:** Department of Anesthesiology, The Second Affiliated Hospital of Jiaxing University, Jiaxing, China

**Keywords:** artificial intelligence, insertion timing, painless gastroscopy, perfusion index, sedation monitoring, ultrasound vocal cord assessment

## Abstract

Painless gastroscopy demonstrates significant improvement in patient comfort and examination compliance, which might indicate that the procedure has become commonly used in clinical practice. However, the critical step of accurately determining insertion timing could present challenges, given that unified standards and objective criteria appear to be lacking. Moreover, improper timing of insertion may increase patient discomfort and the risk of procedural complications while potentially affecting diagnostic accuracy. Traditionally, the eyelash reflex, respiratory patterns, blood pressure, heart rate, and other vital sign changes have been observed by clinicians to assist in judging optimal insertion time. Nevertheless, these indicators show limitations in sensitivity and specificity. Given that rapid advances in anesthesia monitoring technologies have occurred, advanced tools such as the Bispectral Index (BIS), capnography, perfusion index (PI), and ultrasound measurement of vocal cord angles could demonstrate gradual application in assessing insertion timing for painless gastroscopy. Thus, relevant literature may be systematically reviewed in this article. Furthermore, the theoretical foundations and clinical applications of these monitoring indicators might be deeply analyzed. Therefore, the findings could provide clinicians with scientific, objective, and effective methods for evaluating insertion timing, which may enhance safety and efficacy of painless gastroscopy procedures.

## 1 Introduction

Currently, there is no universally accepted or standardized criterion for determining the optimal timing of gastroscope insertion during painless gastroscopy. Gastroscopy demonstrates that it provides an indispensable diagnostic tool for gastrointestinal diseases, and the procedure may be widely used for screening gastric cancer and evaluating significant clinical conditions such as gastritis, ulcers, and other relevant upper gastrointestinal lesions (1, 2). However, traditional gastroscopy performed in the awake state might indicate difficulties such as patient nausea, throat discomfort, and even fear or resistance. Moreover, these factors could lead to procedural challenges, interruptions, and missed diagnoses. Nevertheless, painless gastroscopy has emerged to improve patient compliance and examination quality (3, 4). Painless gastroscopy combines gastroscopic examination with intravenous sedation, inducing a light sleep state in patients. This approach significantly enhances patient experience, shortens procedure time, reduces complications related to reflex movements, and improves clinical compliance (5, 6).

However, the safety and efficacy of painless gastroscopy might largely depend on the timing of endoscope insertion (7). Furthermore, premature insertion before adequate sedation depth could trigger coughing, vomiting, or even aspiration, potentially causing serious complications (8). Thus, delayed insertion may result in over-sedation. Additionally, improper insertion timing can cause reflexive patient movements that hinder endoscopic manipulation and compromise lesion visualization and diagnostic accuracy (9, 10). Nevertheless, insertion timing could mainly rely on the operator’s clinical experience and observations of important external patient signs such as eyelash reflex, verbal responses, and heart rate changes (11–13). In light of these findings, judgments appear subjective and vary among individuals. Therefore, these methods may fail to meet precision and safety requirements of modern painless gastroscopy. Moreover, exploring and integrating an objective system for insertion timing might have become a focal point in clinical practice. Thus, research may establish a real-time evaluation system.

In recent years, with rapid advances in anesthesia monitoring and imaging technologies, tools such as bispectral index (BIS) (14), perfusion index (PI) (15), capnography, and ultrasound evaluation of vocal cord angles have been increasingly applied to monitor anesthesia depth. These modalities provide objective information on sedation level, circulatory status, and airway patency, and may contribute to the development of standardized protocols for determining optimal insertion timing, thereby enhancing procedural safety and diagnostic efficiency.

This narrative review aims to summarize the current evidence regarding traditional clinical indicators and advanced monitoring tools, identify the limitations of existing approaches, and explore potential strategies for optimizing insertion timing in painless gastroscopy. The review was conducted based on a literature search in PubMed, Web of Science, and Embase using relevant keywords related to painless gastroscopy, sedation monitoring, and insertion timing.

## 2 Traditional methods and their limitations

Currently, clinical practice largely relies on the operator’s experience and the patient’s external signs to judge the timing of insertion. However, such subjective judgments exhibit considerable individual variability and lack reproducibility and standardization. In addition, traditional physiological monitoring methods (such as blood pressure and heart rate) are insensitive to the depth of sedation and airway responses, making it difficult to meet the requirements of precise operations. Therefore, it is necessary to introduce more objective assessment tools to assist in determining the timing of insertion.

### 2.1 Eyelash reflex

The eyelash reflex is a classic indicator used to assess light anesthesia. When the patient is in a relatively shallow anesthetic state, gently touching the eyelashes with a cotton swab can trigger a slight eyelid closure response, indicating the presence of some degree of consciousness or brainstem reflex activity (16, 17). Clinically, the eyelash reflex is often used as a preliminary assessment to determine whether the patient has reached the anesthesia depth required for intubation, surgery, or examination (18).

In painless gastroscopy procedures, properly timing the insertion is crucial for reducing patient stress responses, minimizing intraoperative coughing, and decreasing gastroscope intolerance events. Using the eyelash reflex as an evaluation criterion, disappearance of this reflex suggests the patient has entered a sufficiently deep anesthetic state, facilitating safe and smooth gastroscope insertion. This method is simple, intuitive, and requires no instrument assistance, making it especially suitable for clinical settings with limited equipment or where rapid assessment is needed. In clinical practice of painless gastroscopy, the disappearance of the eyelash reflex has been widely adopted as a marker for initiating gastroscope insertion in multiple observational studies. These studies generally indicate that using this criterion is associated with a lower incidence of intraoperative movement and higher endoscopist satisfaction (19).

However, some studies have suggested that relying solely on the disappearance of the eyelash reflex as the indicator for gastroscope insertion may be insufficient. In certain patients, even after the eyelash reflex has disappeared, stronger pharyngeal stimulation (such as passage of the gastroscope) may still provoke severe coughing or unpredictable body movements (20). As a single reflex indicator, the eyelash reflex is susceptible to individual variability and subjective influence by the operator, resulting in relatively limited specificity and sensitivity. Research by Li et al. (7) indicates that in gastroscopy anesthesia protocols using fentanyl combined with target-controlled infusion of propofol, the target propofol concentration should be set at twice the concentration when the eyelash reflex disappears, implying that the anesthetic depth at eyelash reflex disappearance is insufficient to completely suppress the stress response caused by gastroscope insertion. Other studies have shown that inserting the gastroscope 30 s after the disappearance of the eyelash reflex can reduce the incidence of adverse reactions, decrease the propofol dose, and shorten patient postoperative recovery time (21). The subjectivity and delay in judging the disappearance of the eyelash reflex remain unresolved.

### 2.2 Hemodynamic parameters

In clinical practice, after anesthesia induction, if the patient’s blood pressure and heart rate gradually stabilize—particularly if they show a certain degree of decline compared to baseline values (e.g., a 10–20% decrease in systolic blood pressure and heart rate returning to or slightly below baseline)—it usually indicates that the patient has entered a relatively stable anesthetic state (22). During this period, sympathetic nervous system activity is reduced and the stress response is controlled, making it a potentially appropriate time for gastroscope insertion (23). Conversely, if blood pressure and heart rate remain elevated or fluctuate significantly after induction, this often suggests inadequate anesthetic depth, and insertion may trigger adverse events such as coughing, agitation, or involuntary movements. In addition, heart rate variability (HRV) has also been used to assess the depth of anesthesia. By analyzing HRV, information about autonomic nervous system activity can be obtained, thereby indirectly reflecting anesthetic depth (24).

Although hemodynamic indicators such as blood pressure and heart rate are commonly used in clinical settings to assess anesthetic depth, they could reasonably indicate indirect measures that may be influenced by various significant factors. However, these important factors include pre-existing conditions (such as hypertension or arrhythmias), emotional stress, and individual variability in drug sensitivity, all of which might indicate inaccurate assessments (25–27). Moreover, changes in hemodynamic parameters are relatively delayed and may not promptly reflect the level of cortical suppression. Thus, relying on hemodynamic parameters alone to determine anesthetic depth appears to have inherent limitations.

### 2.3 Respiratory pattern changes

During anesthesia induction, as the effect of sedative agents deepens, patients typically undergo a series of changes in spontaneous respiratory patterns. Given that these changes occur, they are usually characterized by a transition from regular, deep, and even breathing to shallower, slower, and less rhythmic respiration. Nevertheless, changes may even include brief periods of apnea. When patients exhibit regular and slow spontaneous breathing—typically at a rate of 10–16 breaths per minute—accompanied by significantly reduced muscle tone, absence of laryngeal reflexes or body movements, and attenuated stress responses, this might suggest an ideal window for gastroscope insertion.

It is important to note, however, that the respiratory patterns could indicate sedation depth yet might be influenced by various factors, including the significant individual variability, the critical type and dosage of anesthetic agents, and the relevant baseline pulmonary function. Airway events following sedation may further confound the assessment of respiratory patterns. Partial upper airway obstruction (e.g., tongue fall) is common under sedation, and may present as snoring or paradoxical thoracoabdominal movement, which can be misinterpreted as deep, slow breathing indicative of adequate sedation (28). If operators rely on these signs to judge sufficient sedation and proceed with gastroscope insertion, this may increase the risk of severe adverse events such as complete airway obstruction or laryngospasm.

Therefore, in clinical practice, reliance on respiratory changes alone to determine timing of insertion should be avoided.

### 2.4 Operator experience

In painless gastroscopy, the endoscopist’s experiential judgment may suggest optimal timing for insertion and has long been widely used. Given that advanced monitoring tools are lacking or resources are limited in clinical settings, the operator’s clinical intuition and experience appear to provide key basis for decision-making.

Experienced endoscopists could more accurately assess whether sedation depth is appropriate for intubation by observing the subtle physiological changes in patients, such as the significant facial expressions, the important muscle tone, the relevant eyelash or eyelid reflexes, the critical respiratory rhythm, and limb movements. Moreover, a relaxed facial expression without frowning or resistance, complete muscle relaxation, and absence of stress responses to external stimuli often indicate ideal time for insertion.

Furthermore, operators also adjust timing based on experience with drug onset, such as observing consciousness changes 30–60 s after propofol administration and combining this with previous procedural responses to flexibly decide insertion moment. Notwithstanding its practical convenience and usability, this “experience-based judgment” appears particularly useful when individual differences are significant or when monitoring equipment is delayed or inaccurate. In such cases, experiential judgment may play important supplementary role.

However, experience-based judgment is inherently subjective and uncertain. It might be influenced by operator’s skill level, clinical pressures, and patient characteristics, lacking objective and quantifiable standards, which could limit generalizability and reproducibility.

## 3 Application of advanced monitoring tools in clinical practice

In light of the advancement of medical technology, a variety of sophisticated monitoring tools have been developed and applied during painless gastroscopy to assist in assessing the significant level of sedation, the important ventilation status, and the critical airway patency.

### 3.1 Application of bispectral index (BIS)

The BIS may be considered an objective, quantitative tool that is used to examine the depth of sedation or anesthesia through analyzing changes in electroencephalographic (EEG) signals. Moreover, the BIS value could range from 0 to 100, with lower values indicating deeper levels of sedation or anesthesia. In painless gastroscopy, BIS monitoring is widely employed because it provides real-time insights into cortical suppression levels, thus aiding in regulation of sedative dosage and determination of appropriate insertion timing.

However, BIS monitoring is considered helpful in optimizing sedation strategies. Additionally, BIS monitoring may reduce drug consumption and minimize sedation-related adverse events. A systematic review and meta-analysis indicated that BIS can significantly reduce total dose of propofol used during gastroscopy and may also shorten recovery time to some extent (29). Nevertheless, another study reported that BIS monitoring could lower incidence of intraoperative hypoxemia, although this finding still requires validation through larger, high-quality randomized controlled trials (30).

Given that BIS values may demonstrate significant potential benefits, determining the appropriate timing for endoscope insertion through BIS monitoring could provide important clinical advantages. Moreover, when BIS values might fall to a certain threshold (e.g., between 60 and 70) and remain stable, it often indicates patients have reached adequate level of sedation. Thus, BIS values can serve as reference point for insertion. However, BIS monitoring may improve both safety and procedural success.

Furthermore, studies have shown that the significant BIS monitoring could enhance both patient and operator satisfaction with these important clinical outcomes. Additionally, one study found that endoscopists using BIS monitoring might report higher satisfaction with sedation quality (31), Thus, BIS also helped reduce sedation-related complications such as hypoxemia, hypotension, and respiratory depression (32). However, some research suggests that while BIS may reduce need for additional propofol, it does not significantly lower overall incidence of sedation- or procedure-related complications (33). Notwithstanding these mixed findings regarding complication rates, the important BIS monitoring could serve as an objective and quantifiable method for assessing sedation depth in painless gastroscopy procedures. Moreover, BIS monitoring might aid in optimizing drug administration and determining optimal insertion timing. Thus, BIS monitoring may enhance procedural safety.

### 3.2 Capnography

Capnography is a non-invasive tool that evaluates ventilatory status by analyzing changes in end-tidal carbon dioxide (EtCO₂) concentration. It provides real-time insights into a patient’s respiratory status and has been widely used in anesthesia and emergency care settings, particularly for assessing sedation depth and assisting in the timing of gastroscope insertion during painless gastroscopy.

Under sedation, continuous monitoring of EtCO₂ enables early detection of respiratory depression or apnea, thereby enhancing patient safety and helping to prevent hypoxia or aspiration-related complications (34). Dynamic observation of the EtCO₂ waveform—when it is stable, regular, and within the normal range (typically 35–45 mmHg)—indicates a relatively steady sedation level, reduced muscle tone, and diminished airway reflexes, making it an ideal window for gastroscope insertion (35).

Conversely, an irregular EtCO₂ waveform, a sudden drop in frequency, or waveform disappearance may indicate either light sedation (with strong airway reflexes still present) or excessive sedation (with respiratory depression or apnea), both of which are unsuitable for insertion (36). Thus, capnography offers endoscopists a dynamic and objective reference, helping to avoid coughing, body movement, and other adverse events during the insertion process.

Capnography, as a real-time and non-invasive method for evaluating respiratory function, enables continuous monitoring of end-tidal CO₂ (EtCO₂) and allows early detection of respiratory depression. Compared with visual assessment of respiratory patterns, capnography is more sensitive and may identify respiratory compromise before clinical signs become apparent. This facilitates more accurate determination of the optimal insertion timing, thereby enhancing procedural safety and efficiency and reducing the risk of sedation-related complications (37).

### 3.3 The role of perfusion index (PI)

The PI is a non-invasive parameter derived from pulse oximetry, calculated based on the absorption of near-infrared light at a wavelength of 940 nm by hemoglobin. It represents the percentage of pulsatile blood flow signal relative to the non-pulsatile component (38). PI dynamically reflects peripheral tissue perfusion and is regulated by sympathetic nervous activity, making it clinically valuable in assessing sedation depth and determining the appropriate timing for endoscope insertion (15, 39).

During sedation, reduced sympathetic activity leads to peripheral vasodilation and an increase in PI. Conversely, when sedation is inadequate or during the recovery phase, heightened sympathetic tone causes vasoconstriction and a decrease in PI. This physiological response enables PI to serve as a reliable indicator of sedation depth (15, 40, 41). Studies have shown that when PI reaches a certain threshold and remains stable, it indicates a sufficiently deep and steady level of sedation, during which muscle relaxation and diminished airway reflexes make it an optimal time for endoscopic insertion (42). Compared with traditional vital signs such as heart rate and blood pressure, PI demonstrates greater sensitivity and faster responsiveness to changes in sedation depth, thereby improving the accuracy of timing judgments and reducing complications such as coughing, movement, and hypoxia due to improper insertion (43).

Furthermore, PI is widely used in anesthesia depth monitoring. In pediatric patients undergoing sevoflurane anesthesia, a strong negative correlation has been observed between PI and the A-line Autoregression Index (AAI), allowing effective tracking of anesthesia depth (15). Another study reported that a threefold increase in PI from baseline serves as a reliable indicator for laryngeal mask airway (LMA) insertion, suggesting that PI can also aid in determining the timing for other invasive procedures (42). Under dexmedetomidine (DEX) sedation, PI has also been shown to predict blood pressure fluctuations, highlighting its versatility in hemodynamic monitoring (44).

In the context of painless gastroscopy, PI serves as a valuable tool for assessing sedation depth and determining the ideal time for scope insertion. Its non-invasiveness, real-time availability, and high sensitivity make it particularly suitable for use in endoscopy centers and various levels of healthcare institutions (43, 45). Incorporating PI monitoring into sedation management protocols can help optimize sedation strategies, enhance procedural safety and efficiency, reduce adverse events, and improve patient comfort and cooperation.

The PI reflects peripheral perfusion and may serve as an indirect indicator of analgesia and autonomic response. As a non-invasive and sensitive parameter, it provides additional information beyond conventional vital signs and may aid in determining optimal insertion timing. However, its clinical utility requires further validation.

### 3.4 Ultrasound assessment of vocal fold angle

Ultrasound assessment of vocal fold angle is an emerging technique that is gaining increasing attention due to its non-invasiveness, real-time capability, and dynamic visualization, offering considerable clinical potential. It enables effective evaluation of vocal fold abduction and adduction angles, which is valuable for assessing the depth of anesthesia and determining the optimal timing for painless gastroscopy insertion.

Several studies have validated the application of ultrasound in vocal fold assessment. For instance, ultrasound has been shown to accurately assess vocal fold movement with high concordance to traditional laryngoscopy findings (46). Moreover, it can dynamically monitor changes in vocal fold angle during anesthesia, providing a useful indicator of sedation depth (47).

In the context of painless gastroscopy, ultrasound assessment of vocal fold angle has demonstrated promising prospects. Accurately determining vocal fold status can help identify the ideal time for scope insertion, thereby enhancing procedural safety and patient comfort (48). Studies have shown that ultrasound serves as an effective tool for real-time monitoring of vocal fold motion, facilitating more precise clinical decision-making (49).

Additionally, ultrasound offers distinct advantages in evaluating vocal fold dysfunction. It enables noninvasive assessment of vocal fold mobility, which is crucial for the early diagnosis and management of postoperative vocal fold paralysis (50). Evidence suggests that the accuracy of ultrasound in assessing vocal fold function is comparable to laryngoscopy, with greater patient acceptance (51). Ultrasound-based vocal cord assessment allows visualization of airway dynamics and may help evaluate airway patency during sedation. This technique provides a direct assessment compared with indirect clinical observation, but its application is operator-dependent and not yet widely standardized.

## 4 Future perspectives

At present, the application of these advanced monitoring tools in painless gastroscopy appears to remain in its early exploratory phase. However, BIS and PI may be increasingly used in clinical practice to guide the sedation and assess the optimal timing of endoscope insertion, though the overall evidence base could indicate limited support. Ultrasound-based vocal cord angle assessment, as a novel, non-invasive, real-time, and dynamic technique, has also attracted attention for potential utility in evaluating sedation depth. Nevertheless, clinical implementation is hindered by challenges such as lack of standardized thresholds and inconsistent measurement protocols. Therefore, future research should focus on development of standardized, multicenter, and large-sample protocols to clarify sensitivity and specificity of these monitoring modalities.

Moreover, with continuous evolution of precision medicine and advancement of intelligent healthcare systems, the assessment of insertion timing during painless gastroscopy may be expected to progress toward multidimensional integration and intelligent decision-making. Given that reliance on a single parameter could often lead to insufficient sensitivity or specificity, the significant integration of these multiple physiological indicators—such as BIS, PI, capnography, and ultrasound-based vocal cord angle assessment—into a composite decision-making framework might reasonably be expected to enhance the clinical accuracy and practicality. Furthermore, application of artificial intelligence in image recognition and physiological signal analysis may offer potential for real-time predictive alerts and procedural guidance. Thus, this could improve precision and safety of endoscopic operations. Additionally, it appears essential to establish standardized operating procedures and interpretation protocols to ensure reproducibility and inter-institutional consistency. In light of these developments, the targeted training programs for endoscopists and anesthesiologists might be necessary to enhance familiarity with emerging monitoring technologies and promote effective integration into routine clinical workflows.

## 5 Limitations

Although several studies have explored the use of objective monitoring tools such as BIS and capnography during painless gastroscopy, direct comparisons with traditional clinical indicators remain limited, and the results are heterogeneous. Furthermore, while combining empirical clinical signs with objective monitoring appears promising, no standardized protocol or consensus has been established regarding the optimal combination strategy. Therefore, current clinical practice still largely depends on clinician experience, and further well-designed prospective studies are needed to establish evidence-based criteria for optimal insertion timing.

## 6 Conclusion

The accurate timing of endoscope insertion could represent a critical determinant of procedural safety and patient comfort in painless gastroscopy procedures. However, the objective monitoring tools such as BIS, PI, and ultrasound-based vocal cord angle assessment may provide promising alternatives to traditional subjective judgment. Moreover, these tools might enable more scientific determination of sedation adequacy. Given that current evidence suggests potential benefits, future efforts may prioritize development of standardized protocols. Nevertheless, multicenter validation studies could promote clinical integration of these technologies.
